# Large Unilamellar Vesicles of Phosphatidic Acid Reduce the Toxicity of α-Synuclein Fibrils

**DOI:** 10.1021/acs.molpharmaceut.3c01012

**Published:** 2024-02-19

**Authors:** Abid Ali, Aidan P. Holman, Axell Rodriguez, Kiryl Zhaliazka, Luke Osborne, Dmitry Kurouski

**Affiliations:** † Department of Biochemistry and Biophysics, Texas A&M University, College Station, Texas 77843, United States; ‡ Department of Entomology, Texas A&M University, College Station, Texas 77843, United States; § Department of Biomedical Engineering, Texas A&M University, College Station, Texas 77843, United States

**Keywords:** α-synuclein, phosphatidic acid, fibrils, AFM-IR, LDH

## Abstract

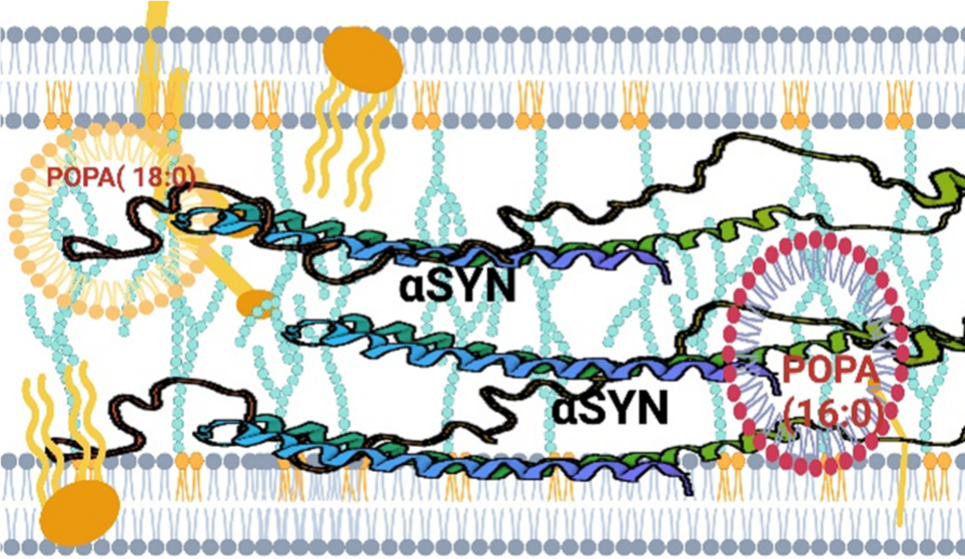

Parkinson’s disease (PD) is a severe pathology that is caused by a progressive degeneration of dopaminergic neurons in substantia nigra pars compacta as well as other areas in the brain. These neurodegeneration processes are linked to the abrupt aggregation of α-synuclein (α-syn), a small protein that is abundant at presynaptic nerve termini, where it regulates cell vesicle trafficking. Due to the direct interactions of α-syn with cell membranes, a substantial amount of work was done over the past decade to understand the role of lipids in α-syn aggregation. However, the role of phosphatidic acid (PA), a negatively charged phospholipid with a small polar head, remains unclear. In this study, we examined the effect of PA large unilamellar vesicles (LUVs) on α-syn aggregation. We found that PA LUVs with 16:0, 18:0, and 18:1 FAs drastically reduced the toxicity of α-syn fibrils if were present in a 1:1 molar ratio with the protein. Our results also showed that the presence of these vehicles changed the rate of α-syn aggregation and altered the morphology and secondary structure of α-syn fibrils. These results indicate that PA LUVs can be used as a potential therapeutic strategy to reduce the toxicity of α-syn fibrils formed upon PD.

## Introduction

Phospholipids constitute a significant portion of plasma and organelle membranes. These lipids have two fatty acids (FAs) esterified to glycerol at the *sn*-1 and *sn*-2 positions, which possesses a phosphoric acid residue at *sn*-3. Unlike other phospholipids, PA does not have a headgroup linked to the phosphoric acid residue.^1,2^ This structural difference alters the lipid geometry, making PA localization highly favorable in highly curved areas of the plasma membrane.^1,2^ Hence, it is expected that PA-rich domains control membrane fusion and fission steps, which are highly important for cell vesicle trafficking.^3^ Furthermore, aging cells are more acidic than younger cells due to higher amounts of anionic phospholipids, such as PA.^4−6^ Therefore, PA and other anionic lipids can play an important role in neurodegenerative pathologies, such as Alzheimer’s (AD) and Parkinson’s diseases (PD).^7−10^

α-Synuclein (α-syn) is a small membrane protein that regulates cell vesicle trafficking (Figure S1).^11,12^ Although intrinsically disordered, α-syn adopts an α-helical conformation in the presence of lipid membranes. An abrupt aggregation of this protein in the midbrain, hypothalamus, and thalamus is linked to PD. This progressive disorder is projected to strike 12 million people by 2040 worldwide.^13^ Current PD treatments focus on mitigating the motor dysfunction caused by the pathology and are not neuroprotective.^14,15^ Therefore, substantial efforts have been made to find therapeutic approaches that can be used to decelerate protein aggregation or lower the toxicity of α-syn fibrils.

Ramamoorthy’s group discovered that lipid vesicles formed by zwitterionic lipids, such as phosphatidylcholine, could lower the toxicity of amyloid fibrils.^16^ It was hypothesized that protein aggregates adsorb to the surfaces of these vesicles, which ultimately minimizes their uptake by the cells. Our group demonstrated that zwitterionic large unilamellar vesicles (LUVs), if were present at equimolar concentrations, inhibited aggregation of α-syn, as well as other amyloidogenic proteins, such as insulin and lysozyme.^17−19^ Recently reported results by Frese and co-workers demonstrated that lipid-altered rates of protein aggregation directly depended on the length and saturation of FAs in phospholipids.^20^ The same conclusion was made by Matveyenka and co-workers about PA that possessed 16:0, 18:0, and 18:1 FAs on insulin aggregation.^21^ Specifically, the researchers demonstrated that 16:0 PA fully inhibited insulin aggregation, whereas 18:1 PA decelerated protein aggregation. It was also shown that 18:0 PA slightly accelerated insulin fibril formation.^21^ Similar experimental findings were reported by Ali and co-workers for transthyretin (TTR). It was found that 18:1 PA accelerated, whereas 18:0 and 16:0 decelerated TTR aggregation.^22^ At the same time, it was found that all forms of PA that were present at the stage of protein aggregation drastically reduced the toxicity of TTR fibrils. The same conclusion was made by Ali and co-workers for lysozyme aggregation in the presence of PA. Specifically, it was found that 16:0 and 18:1 PAs strongly reduced the toxicity of the lysozyme fibrils.

Expanding upon this, we will determine the extent to which PA LUVs with 16:0, 18:0, and 18:1 FAs altered the toxicity of α-syn fibrils. We also utilized a set of biophysical assays to investigate the underlying morphological and structural origin of the observed difference in the toxicity of α-syn/PA fibrils compared with α-syn fibrils formed in the lipid-free environment.

## Experimental Section

### Materials

1,2-Dipalmitoyl-*sn*-glycero-3-phosphate (16:0/16:0-PA, (PA-C_16:0_)), 1,2-dioleoyl-*sn*-glycero-3-phosphate (18:1/18:1-PA, (PA-C_18:1_)), and 1,2-distealoyl-*sn*-3-phosphate (18:0/18:0-PA, (PA-C_18:0_)) were purchased from Avanti (Alabaster, AL).

### Liposome Preparation

To prepare LUVs of PA-C_16:0_, PA-C_18:0_, and PA-C_18:1_, 0.6 mg of each lipid was dissolved in 2.6 mL of phosphate-buffered saline (PBS), pH 7.4. Next, the solution was heated ∼50 °C for 30 min using a water bath. After that, samples were immersed into liquid nitrogen for 3–5 min. The thawing–heating cycle was repeated 10 times. To homogenize the size of lipid vesicles, lipid solutions were passed through a 100 nm membrane using an extruder (Avanti, Alabaster, AL). Finally, we utilized dynamic light scattering to ensure that the size of the LUVs was within 100 ± 10 nm.

### Protein Expression and Purification

The pET21a-α-synuclein plasmid was overexpressed in the *Escherichia coli* BL21 (DE3) Rosetta strain using LB broth media, following the protocol by Volles and Lansbury.^15,31,32^ Two-liter bacterial culture, containing the *E. coliBL21* (DE3) Rosetta strain transformed with the pET21a-α-synuclein plasmid, was cultivated in LB broth media. The culture was induced with 1 mM isopropyl β-d-1-thiogalactopyranoside (IPTG). Following induction, the bacterial cells were separated from the culture medium by centrifugation at 8000 rpm for 10 min. The cell pellet was resuspended in a lysis-tris buffer. This buffer contained 50 mM Tris, 10 mM EDTA, and 150 mM NaCl with a pH of 7.4. A protease inhibitor cocktail from Roche was added to prevent protein degradation. The cell suspension underwent two freeze–thaw cycles and then disrupted the cells and released the protein. The sample was subjected to a 30 min boil in a water bath. Boiling served to denature proteins and aid in releasing the desired protein from cellular components. After boiling, the samples were centrifuged at 16,000*g* for 40 min to separate soluble proteins from cellular debris. For further purification, 10% streptomycin sulfate (136 μL/mL) and glacial acetic acid (228 μL/mL) were added to the supernatant. Subsequently, the mixture was centrifuged at 16,000*g* for 15 min at 4 °C. This step facilitated protein precipitation and removed impurities. The collected supernatant was then precipitated by adding an equal volume of saturated ammonium sulfate at 4 °C. This selective precipitation step aided in further purifying the protein. The precipitated samples were washed with a solution of (NH_4_)_2_SO_4_ at 4 °C, consisting of a mixture of saturated ammonium sulfate and water in a 1:1 v/v ratio. This step contributed to the additional purification of the protein. Ethanol precipitation was repeated twice at room temperature. The collected protein was resuspended in PBS buffer 7.4 and stored at 4 °C for further chromatographic purification.

### Size Exclusion Chromatography (SEC)

α-syn PBS buffer, pH 7.4, was centrifuged for 30 min at 14,000*g* using a benchtop microcentrifuge (Eppendorf centrifuge 5424). The supernatant protein was concentrated using a centricon tube with a 10kd filter. Concentrated protein, 500 μL of synuclein A30P and A53T, was loaded on a Superdex 200 10/300 gel filtration column in AKTA pure (GE Healthcare) FPLC. Proteins were eluted isocratically with a flow rate of 0.5 mL/min at 4 °C using the same buffer, and 1.5 mL fractions were collected according to the ultraviolet–visible (UV–vis) detection at 280 nm. Protein purity was confirmed by the gel (Figure S2).

### α-Synuclein Aggregation

In the lipid-free environment, 100 μM α-syn was dissolved in PBS; the solution pH was adjusted to pH 7.4. For α-syn:PA-C_18:1_, α-syn:PA-C_16:0_, and α-syn:PA-C_18:0_, 100 μM α-syn was mixed with an equivalent concentration of the corresponding LUVs; the pH of the final solution was adjusted to pH 7.4 using the concentrated HCl. Next, samples were placed in a 96 well-plate (SARSTEDT, Nümbrecht, Germany) that was kept in a plate reader (Tecan, Männedorf, Switzerland) at 37 °C for 160 h under 510 rpm agitation. The excitation was set to 450 nm, and the emission was set to 490 nm for ThT kinetics.

### Kinetic Measurements

**Figure 1:**
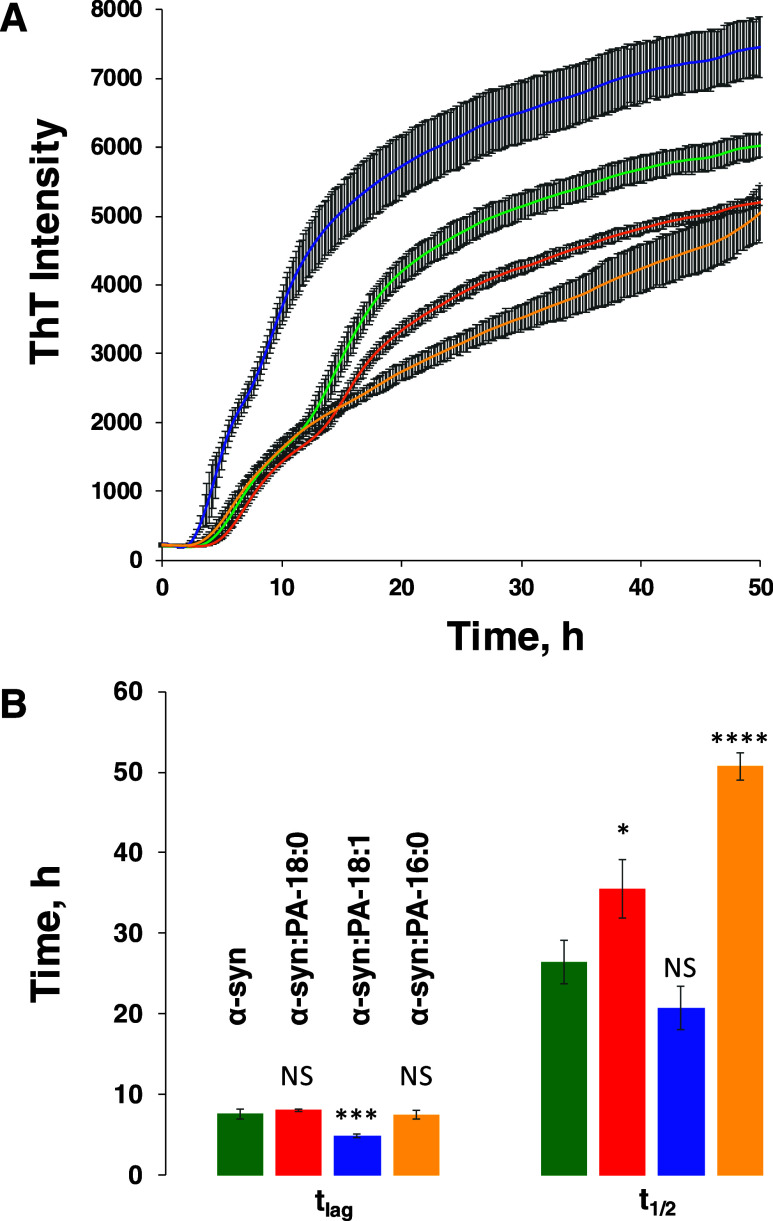
ThT aggregation kinetics (A) with corresponding *t*_lag_ and *t*_1/2_ (B) of α-syn aggregation in the lipid-free environment (α-syn), as well as in the presence of PA-C_18:0_ (α-syn:PA-C_18:0_), PA-C_18:1_ (α-syn:PA-C_18:1_), and PA-C_16:0_ (α-syn:PA-C_16:0_) at 37 °C. According to one-way ANOVA, * *P* < 0.05; *** *P* < 0.001; **** *P* < 0.0001. NS, nonsignificant differences. Standard deviations of three individual repeats are shown in gray.

Rates of protein aggregation were measured using the thioflavin T (ThT) fluorescence assay. For this, samples were mixed with 2 mM ThT solution and placed in a 96 well-plate (SARSTEDT, Nümbrecht, Germany) that was kept in the plate reader (Tecan, Männedorf, Switzerland) at 37 °C for 146 h under 510 rpm agitation (Figure S3). Fluorescence measurements were taken every 10 min (excitation 450 nm; emission 488 nm). Each kinetic curve was an average of three independent measurements. To identify the lag time (*t*_lag_) and half-time (*t*_1/2_) of protein aggregation, ThT intensities at 146 h were normalized to 1. Consequently, *t*_lag_ and *t*_1/2_ reported in Figure 1B indicated 10 and 50% of the maximal ThT intensity, respectively.

### Atomic Force Microscopy (AFM) Imaging

We used an AIST-NT-HORIBA system (Edison, NJ) AFM to perform morphological analysis of protein aggregates. For AFM imaging in light tapping mode, gold-coated force modulation AFM probes (BudgetSensors, Bulgaria, EU) were used. The qualities of a cantilever include a force constant of 1–7 N/m, resonance frequency of 60–90 kHz, and amplitude of 20 nm. For each measurement, an aliquot of the sample was diluted with DI water (20:100 sample–water ratio) and placed on the surface of a precleaned glass coverslip. The dilutions were allowed to dry overnight and washed with DI water to remove salts and the excess sample. Finally, the coverslips were dried under the flow of nitrogen. Preprocessing of the collected AFM images was made using AIST-NT software (Edison, NJ).

### Atomic Force Microscopy Infrared Spectroscopy

Protein samples (3–6 μL) were deposited on a 70 nm gold-coated silicon wafer. After the samples were exposed on the wafer surface for 15–20 min, the excess solutions were removed; the wafers were dried at room temperature. Next, the wafer surface was rinsed with DI water and again dried under N_2_ flow. AFM-IR imaging was conducted using a Nano-IR3 system (Bruker, Santa Barbara, CA). The IR source was a QCL laser. Contact-mode AFM tips (ContGB-G AFM probe, NanoAndMore) were used to acquire the AFM-IR spectra. No evidence of sample distortion was observed upon contact-mode AFM imaging. The contact-mode tip was optimized using a poly(methyl methacrylate) standard sample in 1400–1800 cm^–1^. Totally, 45–50-point spectra were taken, and we can also add that each consists of 3 coaveraged spectra. The region 1648–1654 cm^–1^ was removed to correct the artifact originating from the chip-to-chip transition. The spectral resolution was 2 cm^–1^/pt. Savitzky–Golay smoothing was applied to all spectra with 2 polynomial orders by using MATLAB.

### Circular Dichroism (CD)

After 146 h of incubation at 37 °C, α-syn, α-syn:PA-C_18:1_, α-syn:PA-C_16:0_, and α-syn:PA-C_18:0_ were diluted to the final concentration of 100 μM using PBS and measured immediately using a Jasco J1000 CD spectrometer (Jasco, Easton, MD). Three spectra were collected for each sample within 190–250 nm and averaged.

### Attenuated Total Reflectance Fourier-Transform Infrared (ATR-FTIR) Spectroscopy

After 146 h of incubation at 37 °C, α-syn, α-syn:PA-C_18:1_, α-syn:PA-C_16:0_, and α-syn:PA-C_18:0_ were placed onto the ATR crystal of a 100 FTIR spectrometer (PerkinElmer, Waltham, MA) and dried at room temperature. Three spectra were collected from each sample.

### Cell Toxicity Assays

The rat midbrain N27cells were cultivated on a 96-well plate at a density of 10,000 cells per well, utilizing RPMI 1640 medium (Thermo Fisher Scientific, Waltham, MA), supplemented with 10% fetal bovine serum (Invitrogen, Waltham, MA). This was conducted at a controlled temperature of 37 °C under 5% CO_2_. Post 24 h incubation, the cells exhibited full adherence. Subsequently, 100 μL of the existing culture medium was replaced with an equal volume of RPMI 1640 medium containing a reduced 5% FBS, with 10 μL of the sample. Consequently, the final protein concentration was 10 μM. Following an additional 24 h period of incubation, a lactate dehydrogenase (LDH) assay (CATG1781, Promega, Madison, WI) was used to evaluate the toxicity exerted by the sample. Lysis buffer was used as a positive control. Absorbance readings were recorded using a Tecan plate reader (Mannedorf, Switzerland) at a wavelength of 490 nm.

## Results and Discussion

### Kinetic Studies of α-Syn Aggregation in the Presence of PA LUVs with 16:0, 18:0, and 18:1 FAs

We utilized the ThT assay to examine the extent to which the presence of equimolar concentrations of PA LUVs with 16:0, 18:0, and 18:1 FAs altered the rate of protein aggregation. For this, samples were incubated at 37 °C at 510 rpm shaking in the presence of ThT. We found that in the absence of LUVs, α-syn aggregated with *t*_lag_ = 8.8h ± 0.5h (Figure 1). A similar lag phase was observed for α-syn:PA-C18:0 and α-syn:PA-C16:0. However, we found that PA-C18:1 drastically accelerated primary nucleation of α-syn (*t*_lag_ = 6.0 ± 0.5 h). At the same time, these LUVs did not significantly alter the rate of α-syn aggregation (*t*_1/2_ = 20.0h ± 0.5 h), whereas PA-C18:0 and PA-C16:0 decelerated the rate of fibril formation. Based on these results, we can conclude that the length of FAs and their saturation alter the rate of α-syn primary nucleation (oligomer formation) as well as secondary nucleation that primarily determines oligomer propagation into fibrils.

### Elucidation of the Secondary Structure of α-Syn Aggregates Formed in the Presence of PA-C_16:0_, PA-C_18:0_, and PA-C_18:1_

**Figure 2:**
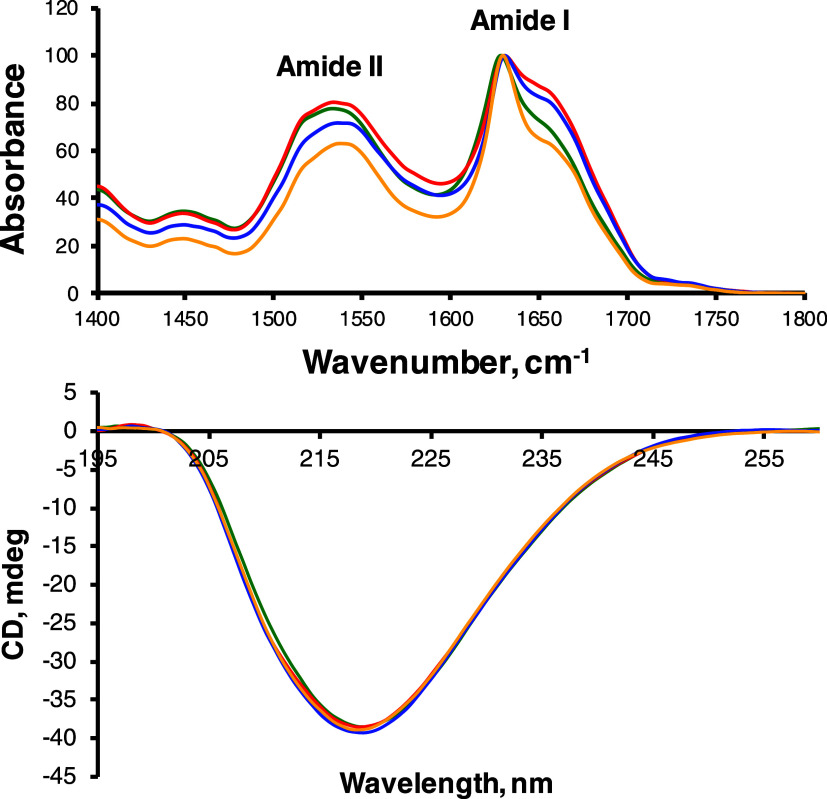
IR (top) and CD (bottom) spectra acquired from α-syn, α-syn:PA-C_18:0_, α-syn:PA-C_18:1_, and α-syn:PA-C_16:0_.

We utilized infrared (IR) spectroscopy and circular dichroism (CD) to determine the secondary structure of α-syn aggregates formed in the lipid-free environment (α-syn) and in the presence of PA-C_16:0_, PA-C_18:0_, and PA-C_18:1_. IR spectra acquired from all samples exhibit both amide I and II bands. The amide I band is centered around 1625 cm^–1^ with a small shoulder ∼1660 cm^–1^, which indicates the predominance of parallel β-sheet with the small contribution of an unordered protein secondary structure in all samples (Figure 2). CD spectra acquired from these samples confirmed the IR results. Specifically, we found that all CD spectra had a minimum at ∼219 nm, indicating the dominance of the β-sheet structure in the protein aggregates formed in the presence and absence of LUVs (Figure 2). Based on these results, we can conclude that the secondary structure of all analyzed protein aggregates is very similar if not identical. Thus, the presence of PA LUVs does not change the secondary structure of α-syn fibrils.

**Figure 3:**
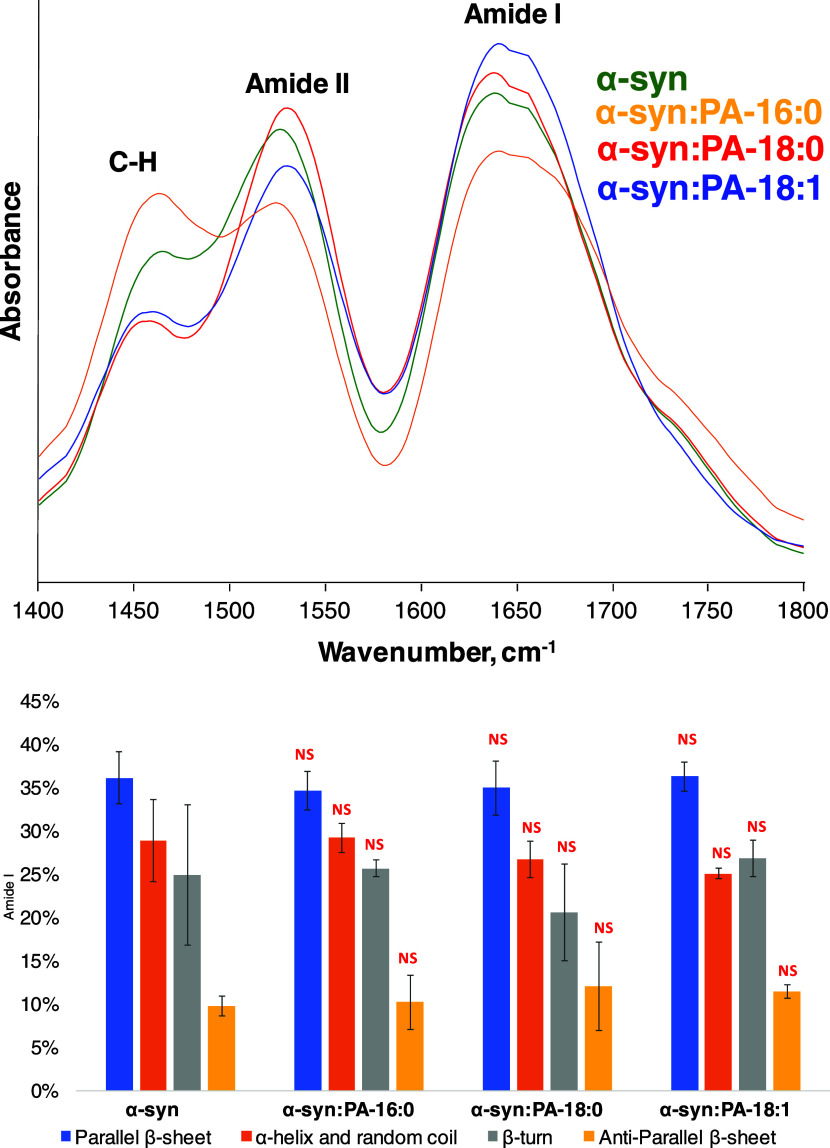
Averaged AAFM-IR spectra (top) acquired from α-syn, α-syn:PA-C_18:0_, α-syn:PA-C_18:1_, and α-syn:PA-C_16:0_. A bar graph (bottom) summarizes the distribution of the protein secondary structure in the protein aggregates according to the fitting of the amide I band. Parallel β-sheet (1624 cm^–1^) in blue, α-helix and random coil (1660 cm^–1^) in orange, β-turn (1675 cm^–1^) in gray, and antiparallel β-sheet (1695 cm^–1^) in yellow.

We also used AFM-IR to examine the secondary structures of individual protein aggregates present in all samples. AFM-IR allows for the direct analysis of such aggregates compared to FTIR or CD, which probes the bulk volume of the analyzed sample. In AFM-IR spectra, we observed amide I and II, as well as C–H vibration (Figure 3).

Next, we fitted amide I to determine the secondary structure of protein aggregates. We found that α-syn, α-syn:PA-C_18:0_, α-syn:PA-C_18:1_, and α-syn:PA-C_16:0_ were dominated by parallel β-sheet with the smaller amounts of α-helix and random coil, as well as β-turn present in their secondary structure. We also found that these protein aggregates had ∼10% of antiparallel β-sheet in their structure. Thus, AFM-IR confirmed the FTIR results discussed above, indicating that the secondary structure of α-syn:PA-C_18:0_, α-syn:PA-C_18:1_, and α-syn:PA-C_16:0_ aggregates was very similar if not identical with the secondary structure of α-syn fibrils.

### Morphology of α-Syn Aggregates Formed in the Presence of PA with Different Length and Saturation of FAs

**Figure 4:**
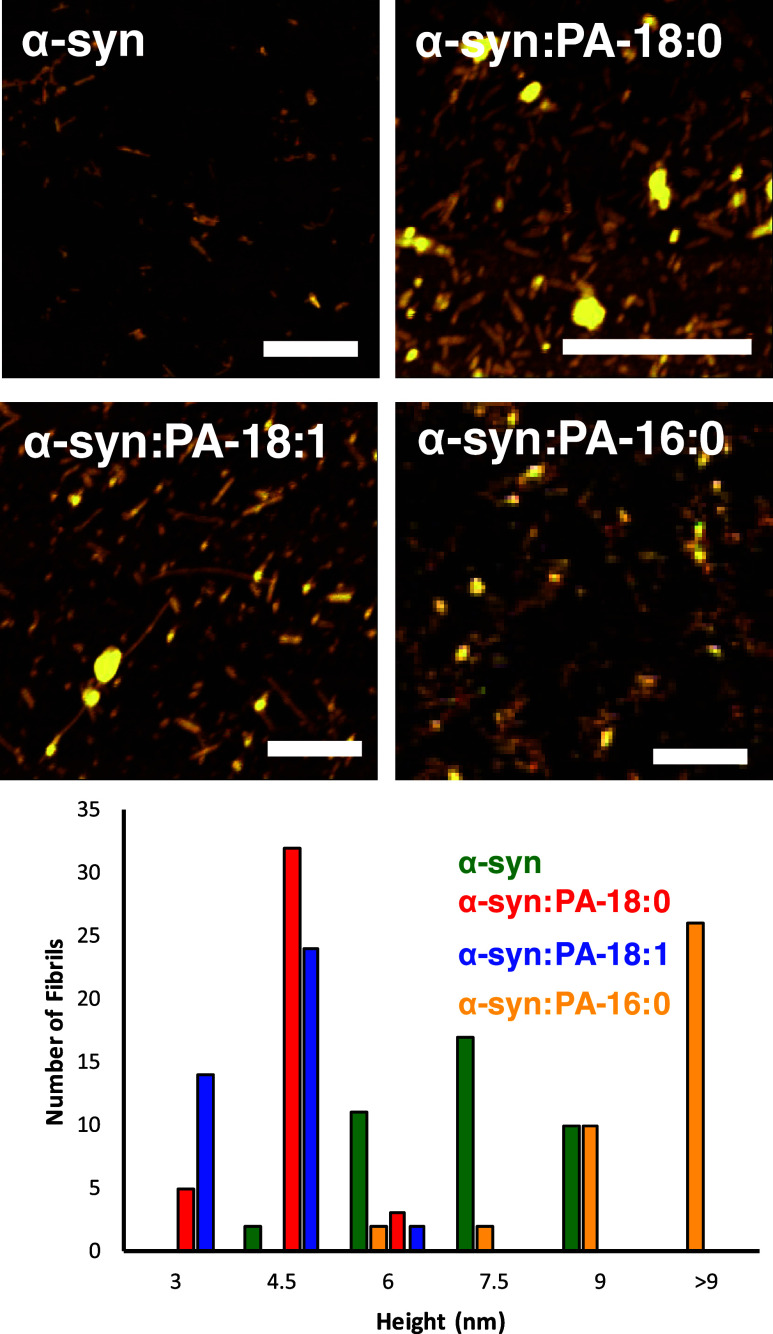
Length and saturation of FAs in PAs alter the morphology of the α-syn aggregates. AFM images (top) and height histograms (bottom) of α-syn fibrillar aggregates formed in the lipid-free environment (α-syn), as well as in the presence of PA-C_18:0_ (α-syn:PA-C_18:0_), PA-C_18:1_ (α-syn:PA-C_18:1_), and PA-C_16:0_ (α-syn:PA-C_16:0_) formed at 37 °C. Scale bars are 500 nm.

In the lipid-free environment, α-syn formed fibrils that were 6 and 9 nm in height (Figure 4). We found that in the presence of both PA-C_18:0_ and PA-C_18:1_, morphologically similar fibrils were grown. However, these fibrils were much thinner (3 and 4 nm in height) than α-syn fibrils formed in the absence of lipids. We also found that in the presence of PA-C_16:0_, α-syn formed spherical oligomers together with short fibrils that had 10–12 nm in height (Figure 4). Thus, we can conclude that the length and saturation of FAs in PA drastically altered the morphology of α-syn aggregates that were grown in their presence.

### Toxicity of α-Syn Aggregates Formed in the Presence of PA with Different Length and Saturation of FAs

**Figure 5:**
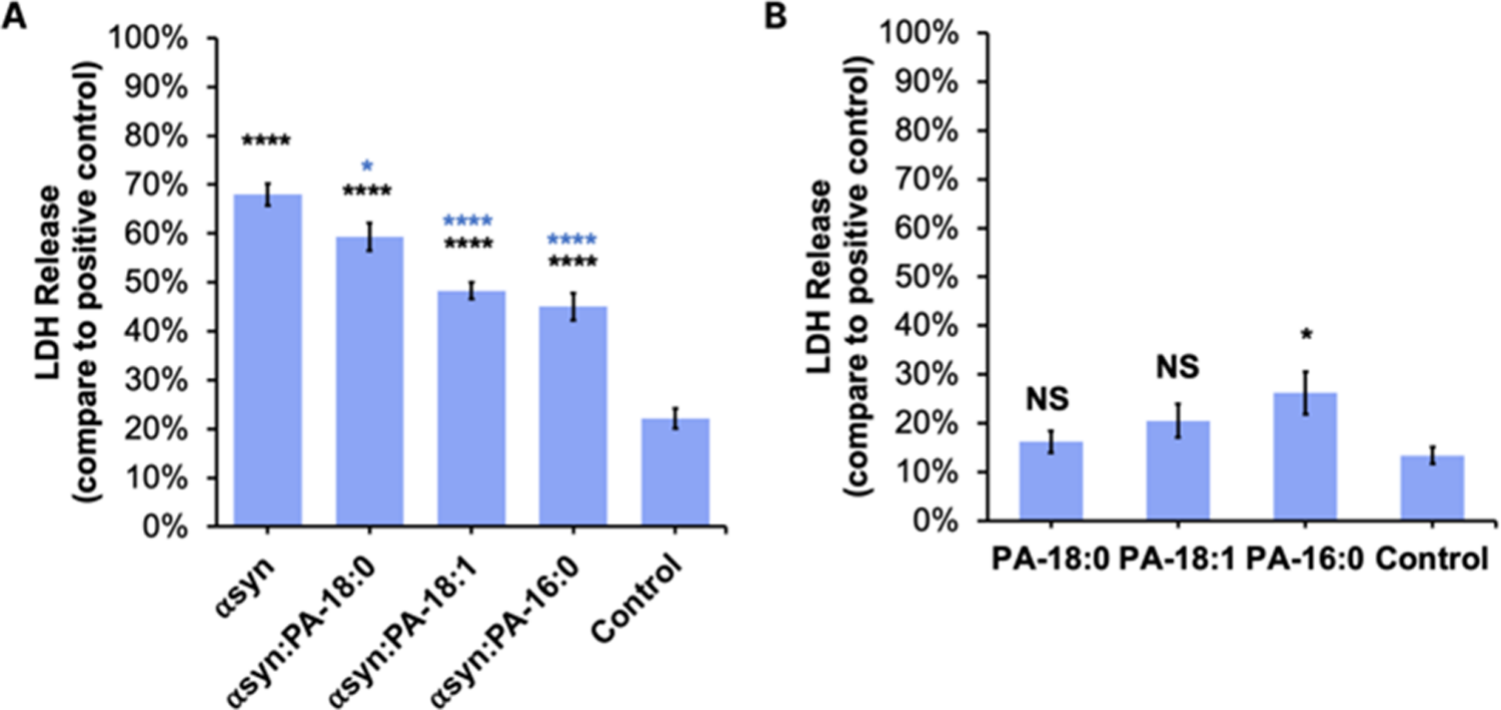
Histograms of LDH assays reveal cell toxicity of (A) α-syn, α-syn:PA-C_18:0_, α-syn:PA-C_18:1_, and α-syn:PA-C_16:0_ and (B) the lipid themselves. Black asterisks (*) show significance level of differences between protein aggregates and the control; blue asterisks show significance level of difference between α-syn and α-syn:PA-C_18:1_, α-syn:PA-C_16:0_, and α-syn:PA-C_18:0_; according to one-way ANOVA, * *P* < 0.05, **P < 0.01, ***P < 0.001, ****P < 0.0001. NS shows the absence of statistical significance between α-syn and α-syn fibrils formed in the presence of LUVs with different PAs. The results of the LDH assay show no cell toxicity for PA-C_18:0_ and PA-C_18:1_, whereas PA-C_16:0_ was only slightly toxic to N27 rat dopaminergic cells (B). Black asterisks show significant levels of differences between LUVs and the control.

The question to ask is whether the observed morphological differences among α-syn, α-syn:PA-C_18:0_, α-syn:PA-C_18:1_, and α-syn:PA-C_16:0_ have any biological significance. To answer this question, we investigate the extent to which these protein aggregates exert cell toxicity to mice midbrain N27 cell line (Figure 5).

LDH assay revealed a statistically significant difference between the toxicity exerted by α-syn and α-syn fibrils formed in the presence of PAs. We also found that α-syn:PA-16:0 aggregates exerted the lowest cell toxicity compared to α-syn:PA-C_18:1_ and α-syn:PA-C_18:0_ fibrils. We also found that PA-C_18:0_ and PA-C_18:1_ LUVs themselves were not toxic, while PA-C_16:0_ LUVs were only slightly more toxic than the control. Based on these results, we can conclude that PA LUVs drastically lower the toxicity of α-syn fibrils.

## Discussion

Lipid membranes play an important role in protein aggregation. Galavagnion and co-workers showed that at low concentrations relative to the concentration of α-syn, LUVs accelerated the rate of protein aggregation.^11,12,23^ Using fluorescence imaging, Hannestad and co-workers demonstrated that the acceleration of α-syn aggregation was caused by strong protein–lipid interactions.^24^ NMR revealed that in such cases, α-syn formed strong electrostatic and hydrophobic interactions with lipids, which resulted in disruption and asymmetric deformation of lipid membranes.^25,26^ Electrostatic interactions were primarily observed between the polar lipid heads and the highly charged N-terminus of α-syn. At the same time, hydrophobic interactions dominated between the central part of the protein and aliphatic tails of fatty acids (FAs) present in such lipids.^27,28^ Numerous studies reported by Claessens’s group indicated that such interactions were directly determined by the charge of lipids and the size of lipid vesicles.^29−35^ These results were confirmed by Frieg and co-workers who used cryo-EM to resolve the secondary structure of α-syn fibrils formed in the presence of lipids.^36^ These researchers also found that phospholipids promoted an alternative protofilament fold, which led to an unusual arrangement of protofilaments and filled the central cavities of the fibrils.

At the same time, Esbjörne’s group showed that amyloidogenic proteins, such as Aβ_1–42_, could simply aggregate on the surfaces of LUVs without a direct interaction with lipids present in the vesicles.^37^ One may expect that such LUV-templated aggregation may yield structurally similar or identical protein aggregates compared to those formed in the lipid-free environment. Furthermore, Ramamoorthy’s group found that the toxicity of LUV-templated fibrils was lower compared to the toxicity of amyloid fibrils formed in the absence of lipids.^16^

Our current results presented here confirm the experimental concept reported by the Esbjörne and Ramamoorthy groups. Specifically, we found that the toxicity of α-syn aggregated grown in the presence of PA LUVs was lower than the toxicity of α-syn fibrils formed in the lipid-free environment. We also found no differences between α-syn fibrils formed in the presence of LUVs with different PAs and under lipid-free conditions. One can expect that the size of amyloid fibrils may determine their penetration properties into cells and consequent cell toxicity. However, our previously reported results indicated that the unlikely morphology of protein aggregates could be the major cause of the observed differences in their toxicity.^22,38,39^ We expect that the differences may arise from the degree of endosomal damage and free radical activity of such fibrils that, as was shown by our group, was difference for insulin fibrils formed in the lipid-free environment and in the presence of lipids.^40^

Similar experimental evidence of lipid-suppressed toxicity was recently reported by our group for TTR and lysozyme.^22^ Specifically, we found that in the presence of PA with different lengths and saturations of FAs, both TTR- and lysozyme-formed fibrils that exerted less cytotoxicity compared to fibrils formed in the lipid-free environment. It should be noted that Matveyenka and co-workers previously demonstrated the cytoprotective properties of PA-C_16:0_ for insulin aggregates, whereas no effect was observed for PA-C_18:0_ and PA-C_18:1_ LUVs.^21^ Thus, these findings demonstrate that PA LUVs could be considered a potential therapeutic platform that can decrease the toxicity of a large spectrum of amyloid aggregates.

If you recall, aging cells are more acidic than younger cells due to higher amounts of anionic phospholipids.^4−6^ Our results indicate that the accumulation of PA, an anionic phospholipid in the plasma membrane, can alter the stability of amyloid proteins and either accelerate or decelerate their aggregation rate. Therefore, it becomes critically important to understand the effect of other anionic lipids on the aggregation of amyloid proteins.

In summary, our results demonstrate that PA LUVs can strongly suppress the cytotoxicity of α-syn fibrils. The magnitude of the suppression of amyloid toxicity has a direct relationship with the length and saturation of FAs in PA. At the same time, these structural differences of PAs had no effect on the secondary structure of α-syn fibrils formed in their presence. We also found that the unsaturation of FAs in PA results in the acceleration of α-syn nucleation. However, unsaturation has no effect on the rate of secondary nucleation, whereas the presence of PA LUVs with both 16:0 and 18:0 decelerated the rate of secondary nucleation of α-syn. Based on these findings, one can expect that *in vitro* made monolipid LUVs can be used as therapeutic platforms to decelerate the progression of PD.

## Supporting Information Available

Sequence of α-syn, a gel image of purified α-syn, and ThT aggregation kinetics of α-syn with standard bars that represent three individual repeats are shown in Figures S1–S3 (PDF)
