# Supplementary material for: Large Unilamellar Vesicles of Phosphatidic Acid Reduce the Toxicity of α-Synuclein Fibrils
Source: Mol Pharm. 2024 Feb 19;21(3):1334–41. doi: 10.1021/acs.molpharmaceut.3c01012 (PMC10915799; doi:10.1021/acs.molpharmaceut.3c01012)
Supplement: Supplementary file 1 — mp3c01012_si_001.pdf [file mp3c01012_si_001.pdf]

Large Unilamellar Vesicles of Phosphatidic Acid Reduce the Toxicity of α-Synuclein Fibrils

Abid Ali,[1] Aidan P. Holman,[1-2] Axell Rodriguez,[1] Kiryl Zhaliazka,[1] Luke Osborne[1] and Dmitry Kurouski[*1,3]

1. Department of Biochemistry and Biophysics, Texas A&M University, College Station, Texas 77843, United States
2. Department of Entomology, Texas A&M University, College Station, Texas 77843, United States
3. Department of Biomedical Engineering, Texas A&M University, College Station, Texas, 77843, United States

Supporting Information

MDVFMKGLSKAKEGVVAAAEKTKQGVAEAAGKTKEGVLYVGSKTKEGVVH

GVATVAEKTKEQVTNVGGAVVTGVTAVAQKTVEGAGSIAAATGFVKKDQL

GKNEEGAPQEGILEDMPVDPDNEAYEMPSEEGYQDYEPEA

Figure S1. sequence of a-synuclein with negatively charged amino acid residues are highlighted by red and positively charged by blue. At neutral pH, the net charge of the protein is -9.

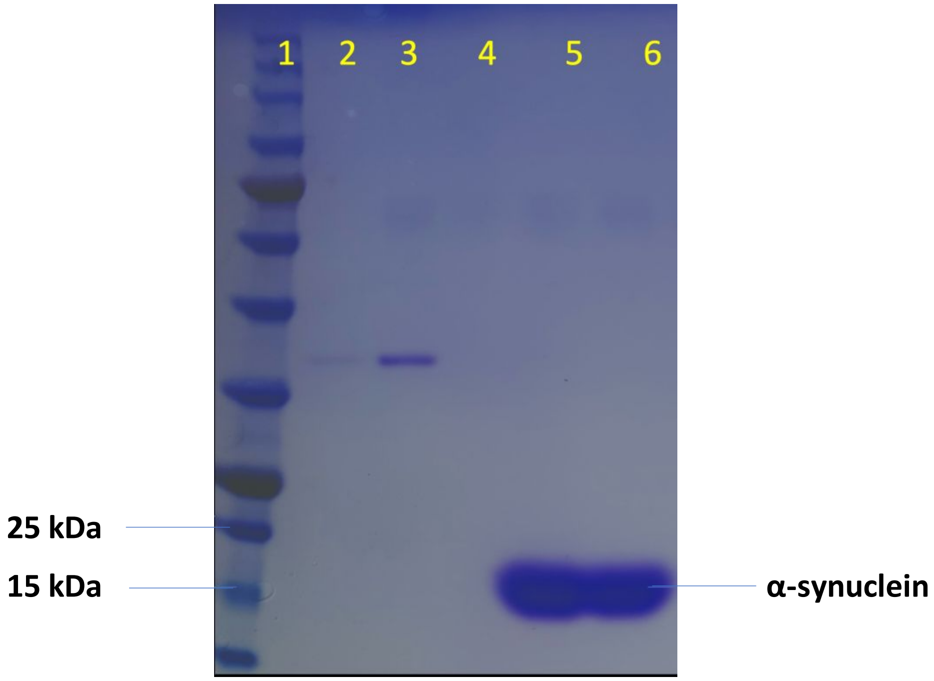

Figure S2. Gel image of purified α-synuclein (5 and 6) together with the wash buffer (2 and 3). Protein latter is in 1.

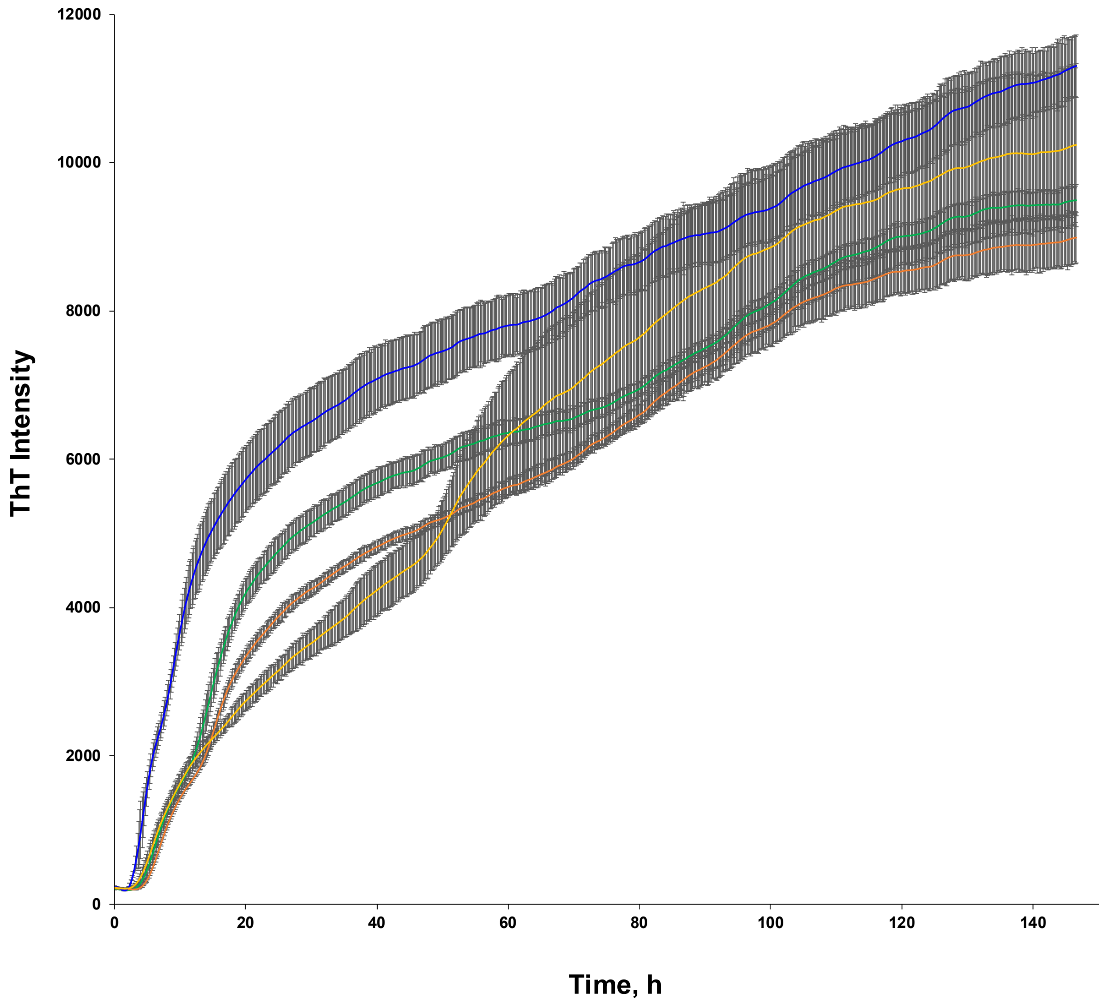

Figure S3. ThT aggregation kinetics of α-syn aggregation in the lipid-free environment (α-syn (green)), as well as in the presence of PA-C18:0 (α-syn:PA-C18:0 (red)), PA-C18:1 (α-syn:PA-C18:1 (blue)) and PA-C16:0 (α-syn:PA-C16:0 (yellow)) at 37 °C. Standard deviations of three individual repeats are shown in grey.
